# P-33. Vaccine Hesitancy in HIV Patients Following the COVID19 Pandemic

**DOI:** 10.1093/ofid/ofae631.240

**Published:** 2025-01-29

**Authors:** Kory Kropman, Amit T Vahia, Susan M Szpunar, Leonard B Johnson

**Affiliations:** Ascension St. John Hospital, Grand Rapids, Michigan; Ascension St. John Hospital, Grand Rapids, Michigan; Ascension St. John Hospital, Grand Rapids, Michigan; Ascension St. John Hospital, Grand Rapids, Michigan

## Abstract

**Background:**

The prevalence of vaccine hesitancy has been rising over the last several years and was exacerbated by the Covid19 epidemic with the introduction of the novel Covid19 vaccines. There are concerns that immunosuppressed individuals such as HIV patients may also demonstrate vaccine hesitancy to recommended non-Covid19 vaccines. In this study, we evaluated the rate of refusal of non-Covid19 vaccines in the pre- and post-period after release of the Covid19 vaccine.

Table 1

**Figure:**
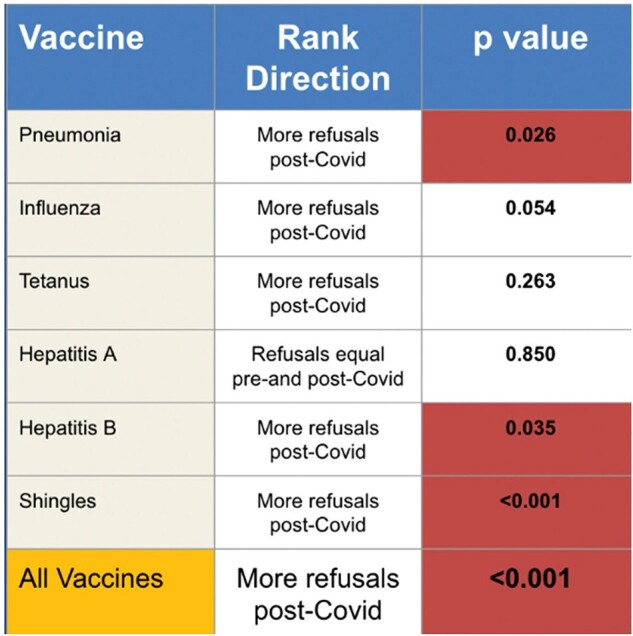

Summary of Vaccination Results. The distribution of the Pre-Covid and Post-Covid Group for each tested vaccine were compared. The direction of the distribution trend is listed and whether or not it was statistically significant (p<0.05)

**Methods:**

We measured and compared the number of vaccines offered and refused at each visit over the total number of visits for 195 outpatients with HIV for 18 months and pre- and post-release of the Covid19 vaccines to the general public. The vaccines studied included the following: tetanus, pneumococcal, shingles, hepatitis A and B, and influenza. The data was collected using hospital records from the electronic medical record as well as Michigan Care Improvement Registry (MICR). Data was analyzed using descriptive statistics and the Wilcoxon signed ranks test. The Mann-Whitney U test, Kruskal-Wallis test and Pearson’s correlation were performed for this vaccine. In all statistical tests, a p-value less than 0.05 was taken to indicate statistical significance.

**Results:**

Collectively, there were more refusals in the period after release of the Covid19 vaccine for non-Covid19 vaccines. This was also the case for all individual vaccines, except hepatitis A. There was an overall statistically significant difference (p < 0.001) in the distribution of vaccine refusals between the pre-Covid19 and post-Covid19 period. This was observed for the pneumococcal (p=0.026), hepatitis B (p=0.035), and shingles vaccine (p < 0.001) respectively.

**Conclusion:**

This study provides evidence that there is an increase in non-Covid19 vaccine hesitancy following the release of the Covid19 vaccine among HIV outpatients. Larger studies should be performed to confirm this observation.

**Disclosures:**

**All Authors**: No reported disclosures

